# Thirteen Years of Phleboviruses Circulation in Lombardy, a Northern Italy Region

**DOI:** 10.3390/v13020209

**Published:** 2021-01-29

**Authors:** Elena Percivalle, Irene Cassaniti, Mattia Calzolari, Davide Lelli, Fausto Baldanti

**Affiliations:** 1 Molecular Virology Unit, Microbiology and Virology Department, Fondazione IRCCS Policlinico San Matteo, 27100 Pavia, Italy; e.percivalle@smatteo.pv.it (E.P.); i.cassaniti@smatteo.pv.it (I.C.); 2 Department of Clinical Surgical Diagnostic and Pediatric Sciences, University of Pavia, 27100 Pavia, Italy; 3 Istituto Zooprofilattico Sperimentale della Lombardia e dell’Emilia Romagna B. Ubertini, 25100 Brescia, Italy; mattia.calzolari@izsler.it (M.C.); davide.lelli@izsler.it (D.L.)

**Keywords:** phleboviruses, epidemiology, Lombardy region

## Abstract

Phleboviruses transmitted by phlebotomine sandflies are endemic in the Mediterranean basin. *Toscana phlebovirus* (TOSV), *Sicilian phlebovirus* (SFSV), and *Naples phlebovirus* (SFNV) are responsible of summer fever, with well-known pathogenic potential for humans ranging from asymptomatic to mild fever, in addition to neuro-invasive infections during summer. Although TOSV, in particular, is a significant and well-known human pathogen, SFVs remain neglected, with many gaps in the relevant knowledge. Sero-epidemiological studies and case reports recently showed a geographical wider distribution than previously considered, although the real incidence of phleboviruses infections in the Mediterranean area is still unknown. Here we retrospectively evaluated the circulation of phleboviruses during summer seasons between 2007 and 2019 in 649 patients showing neurological symptoms using both molecular and serological approaches. We found that 42/649 (6.5%) subjects experienced phlebovirus infection and only 10/42 cases were detected by molecular assays, whereas the other 32/42 were identified using serological approaches, including neutralization assays. During the 2013 summer, an outbreak in the Lombardy region is described because the prevalence of phlebovirus infection reached 37.2% (19/51 subjects). Interestingly, only 5/19 (26.5%) reported traveling in endemic areas. Of note, no cross-neutralization was observed between different strains tested, showing the possibility to be reinfected by newly discovered phlebovirus strains. In conclusion, phlebovirus infections are still inadequately considered by physicians and are generally underestimated. However, based on our results, sandfly fever viruses should be routinely included in diagnostic panels during summer period, including in Northern Italy.

## 1. Introduction

The genus Phlebovirus of the Phenuviridae Family (order Bunyavirales) has been recently expanded and currently includes 60 viral species officially recognized by the International Committee on Taxonomy of Viruses (ICTV) [1].

From a virological point of view, phleboviruses are enveloped viruses characterized by three-segmented, single-stranded, negative-sense RNA with a high rate of point mutations, reassortments, and recombinations that generate new viruses [2]. For this reason, several new phleboviruses have been discovered, increasing the species variety [3]. Although no reservoir host has been definitely identified for phleboviruses, a role of humans as a reservoir has been hypothesized because of a transient viremia in the case of the Toscana virus (TOSV) [4]. Moreover, the high vertical transmission rate allows hypothesizing about the role of reservoirs in the viral cycle for competent sandflies [4].

TOSV, Sandfly fever Sicilian virus (SFSV), Sandfly Naples virus (SFNV), and Sandfly fever Cyprus virus (SFCV) are SFVs with well-known pathogenic potential for humans. In detail, SFSV, SFNV and SFCV are responsible for a self-limited febrile condition, whereas TOSV has been identified as a causative agent of meningitis and mild meningoencephalitis [5,6] in central and southern Italy [6,7,8], Spain [9,10], and southern France [11] during summer. Toscana virus (TOSV) [12] was first isolated from Phlebotomous perniciosus in Central Italy (Tuscany) in 1971 [13]. This virus is widely distributed in the Mediterranean region, Central and Eastern Europe, and North Africa and Turkey, but also in the Indian subcontinent, the Middle East and central Asia [14].

Several new phleboviruses have been described in recent years, including in the Lombardy region, and their pathogenic potential is still uncharacterized. In this setting, Ponticelli viruses, which are included as *Adana* phlebovirus species because they share more than 95% of the identity of the amminoacid sequence of the L segment, have also been identified. The real incidence of phleboviruses infections in the Mediterranean area is still unknown, although data indicating their increasing spread in other European countries have been reported [15].

To date, these viruses have been neglected due to the lack of specific symptoms and the underestimation of their geographical spread. In this retrospective study, we defined the circulation of phleboviruses using both serological and molecular assays during summer seasons between 2007 and 2019 in a group of patients showing neurological symptoms and who were negative for other neurotropic viruses. Moreover, we aimed to evaluate the specificity of neutralizing antibodies against different serotypes of newly discovered phleboviruses, which showed a lack of cross-protection between different strains. Finally, we describe an outbreak that occurred in a non-endemic region (Lombardy, Northern Italy), in 2013, that involved 19 patients, many of whom did not have a travel history in endemic areas, providing a new map of phlebovirus circulation.

## 2. Materials and Methods

### 2.1. Patients

Overall, 649 patients affected by neurological symptoms, ranging from mild (disorientation and confusion) to severe (meningitis, meningoencephalitis), were referred to our reference regional laboratory in the Lombardy region for diagnosis between 2007 and 2019 summer seasons (from June until October). Current phlebovirus infection was defined as following: (1) a positive TOSV real-time reverse transcriptase polymerase chain reaction (RT-PCR) and/or a positive reverse transcriptase (RT) nested-PCR Panphlebovirus independent from serological assay; (2) negative molecular tests but presence of specific phlebovirus IgM, IgG, or neutralization assay (NTA) seroconversion; (3) an increase in NTA titer during the follow-up period.

### 2.2. Molecular Assays

A total of 277 cerebrospinal fluid (CSF) samples from 277/649 (42.7%) patients, negative for neurotropic viruses including HSV, VZV, CMV, EBV, JC/BK virus, HHV-6, B19, enterovirus, parechovirus, and West Nile virus were analyzed for phleboviruses. Two methods for virus detection and identification were used: (i) a specific TOSV real-time reverse transcriptase polymerase chain reaction (RT-PCR) [11] and (ii) a reverse transcriptase (RT) nested-PCR Panphlebovirus able to detect SFNV, SFSV, TOSV, Rift Valley fever virus (RVFV), Aguacate virus (AGUV), and Punta Toro virus (PTV). Sequencing of amplicons was performed by the Sanger method for positive results [16].

### 2.3. Serological Assays

All of the 649 sera from the enrolled patients were tested for phlebovirus serology with an indirect immunofluorescence test (IIFT) for Sandfly fever virus Mosaic 1 types Sicilian, Naples, Toscana, Cyprus IgM and IgG (EUROIMMUN; Lubeck, Germany), which allows for the simultaneous detection of antibodies against SFSV, SFNV, TOSV, and CFCV. In detail, a 1:10 dilution for all sera were first tested and only positive results were titrated with four-fold dilution. For the detection of IgM antibodies, serum samples were previously absorbed at 1:10 dilution with EUROSORB (EUROIMMUN AG, Lubeck, Germany). Briefly, 10 µL of serum sample was added in double to a multiwell slide and incubated for 30 min at 37 °C in CO_2_. After washing, 10 µL of anti-human IgG or IgM fluorescein antibodies (EUROIMMUN AG) were added and incubated again for 30 min at 37 °C in CO_2_. After mounting in glycerol, slides were read under a fluorescent microscope at 10×.

### 2.4. Neutralization Assay

Positive serum samples were also tested with a neutralization assay (NTA) with four phleboviruses including a TOSV strain 181135-14/2013 (GenBank: KU573064-KU573066), and three reassortant phleboviruses, denominated Ponticelli I 181135-4/2013 (GenBank: KX388223-KX388225), Ponticelli II 181135-8/2013 (GenBank: KX388220-KX388222), and Ponticelli III 220116-5/2013 (GenBank: KX388208-KX388210) viruses. All of these strains were isolated from pools of sandflies and kindly provided by Istituto Zooprofilattico Sperimentale “Bruno Ulbertini” of Brescia [17]. Briefly, 50 µL of each serum, starting from 1:10 with a serial four-fold dilution series, were added in two wells of a flat bottom tissue culture microtiter plate (COSTAR, Corning Incorporated, NY 14831, USA), mixed with an equal volume of 50 TCID50 of each Phlebovirus strain previously titrated and incubated at 37 °C in 5% CO_2_. After one hour, 50 µL of 10^4^ Vero cells (VERO C1008 (Vero 76, clone E6, Vero E6); ATCC**^®®^** CRL-1586™) were added to each well and incubated at 37 °C with 5% CO_2_ for 5 days. Wells were scored with an inverted microscope to evaluate the degree of cytophatic effect (CPE) compared to the virus control. The neutralizing titer was the maximum dilution with the reduction of 90% of the cytopathic effect. A positive titer was equal to or greater than 1/10. Positive and negative standards provided by EUROIMMUN and from patients with positive molecular and serology assay were included in all tests.

### 2.5. Ethics Statement

The local Ethics Committee consent was not required because according to a Regional Surveillance and Preparedness Plan (DGR 12591, 27 December 2012). Diagnostic detection of flavivirus and phlebovirus infections in the Lombardy Region was centralized at the Regional Reference Laboratory (Molecular Virology Unit, Fondazione IRCCS Policlinico San Matteo, Pavia). Informed consent was not necessary because patients with suspected infections were included in a regional diagnostic protocol. All the data were analyzed anonymously according to a Regional Surveillance and Preparedness Plan (DGR 12591, 27 December 2012).

## 3. Results

### 3.1. Characteristics and Distribution of Patients Positive for Phleboviruses

**Figure 1:**
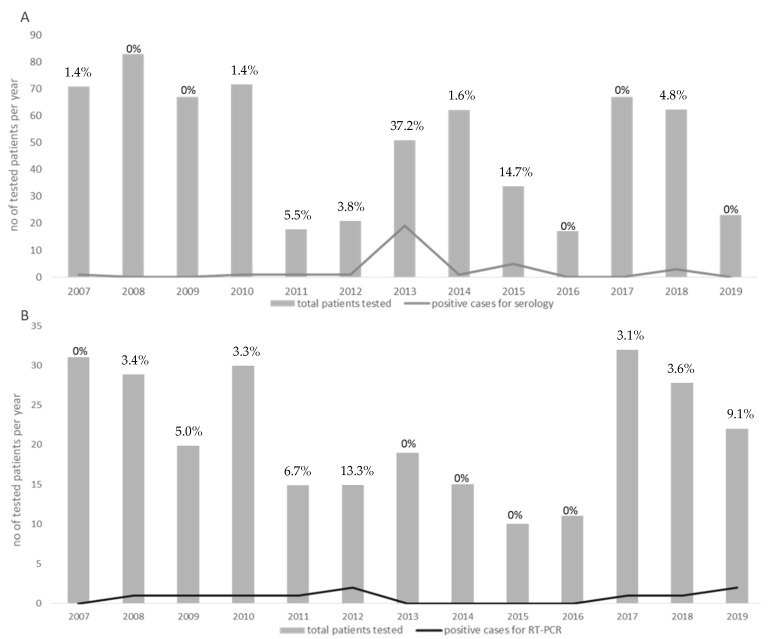
Annual distribution of the total number of patients tested between 2007 and 2019 (grey boxes). The number of positive cases diagnosed during each year is indicated by grey lines. The number of positive cases for serology and for RT-PCR is given in (**A**,**B**), respectively. Percentages of positive cases per each year are given in each graph.

Forty-two patients out of 649 (6.4%) (17 females and 25 males; median age 54.3, range 16–98 years) were defined as positive for phlebovirus infections by molecular and/or serological assays. The distribution of all of the cases reported between 2007 and 2019 was classified according to serology (Figure 1A) and molecular results (Figure 1B). The percentage of positive infected patients ranged from a maximum of 37.2% in 2013 to a lack of cases in 2016. For 17 (40.5%) patients, a history of travels in endemic areas was reported, whereas 17 (40.5%) patients did not report a history of travel in the previous weeks. Finally, for the remaining 8 (19%) patients, no data about travelling in endemic areas were available.

### 3.2. Patterns of Molecular and Serological Diagnosis

Among the 42 diagnosed phlebovirus infections, 10 (23.8%) were detected by the molecular test. In detail, one (2.3%) was detected only by TOSV RT-PCR at the admission (no other samples were available for serology) and nine (21.4%) were detected by a combination of TOSV/Panphlebovirus virus RT-PCR and serology. The remaining 32 infections (76.1%) were identified only by serological assays (Table 1). In Fifteen patients with two consecutive serum samples a seroconversion was observed; in detail, five were TOSV RT-PCR positive patients and 10 were positive only by serology.

### 3.3. Phlebovirus Outbreak in 2013 Reported in Lombardy Region

Of note, in 2013, an outbreak of 19 cases of phlebovirus infection out of 51 patients living in the Lombardy region (37.3%) and referred to our hospital for neurological problems was reported. This cluster involved nine females and 10 males, with a median age of 60 years (range 23–98). Among these patients, only 5 (26.3%) had a history of travel in endemic areas (one in Liguria, two in Southern Italy, one in the Romanian coast, and one in Greece) in the weeks before the onset of symptoms, whereas the other 14 patients (73.7%) had no history of travel, suggesting the local acquisition of the infection. No phleboviruses were detected by molecular tests in plasma samples and in the CSF available. Analyzing serological results at the time of admission, three groups of patients were identified: (i) eight patients (id #1–#5, #11, #13, #17) (42.1%) with positive IgM, and negative IgG and neutralizing antibodies (Nt Abs); (ii) five patients (#10, #14–#16, #18) (26.3%) positive for IgM, IgG, and Nt Abs; and (iii) six patients (#6–#9, #12, #14) (31.5%) positive for both IgM and IgG but negative for Nt Abs (Table 2).

### 3.4. Follow-Up of Seven Patients Revealed Different Serological Pattern of Seroconversion

Follow-up samples were available in seven patients (id #1, #5, #7, #8, #10, #11, and #13) and data of seroconversion are reported in Table 3. In detail, in four patients (#5, #7, #11, and #13), we observed a seroconversion for both IgG and Nt Ab, whereas in patient #1 only a seroconversion for IgG, but not for Nt Abs, was detected. In one patient (#8) with positive results for IgM, IgG, and Nt Abs at the time of admission, IgM was negative in a second sample collected after three weeks, and in patient #10, who reported positive IgM and IgG results, Nt Abs seroconversion was observed. Focusing on neutralizing results reported Table 3, it was also observed that the reaction against the four phlebovirus strains was restricted only to Toscana and Ponticelli II. In particular, serum samples obtained from patients #5, #8, and #13 reacted to TOSV (strain 181135/14) with a Nt Abs titer of 20, 40 and 640 respectively. One sample (patient #13) showed a low cross-reactivity also with Ponticelli II. Three patients (#7, #10 and #11) recognize only Ponticelli II strain with a Nt Abs titer ranging from 10 to 40. No serum samples showed neutralizing activity against the two strains of Ponticelli I and III. The Nt Abs of the patients #14, #15, #16, and #18, for which only one serum sample was available, reacted only with Ponticelli II with Nt Abs titer of 10. 

## 4. Discussion

Phleboviruses represent a genuine concern for residents and travelers because they are etiologic agents of viral meningoencephalitis in the summer season in Mediterranean countries [15]. Although viral RNA detection represents a useful diagnostic tool for identifying acute TOSV-associated central nervous system infections [18], in our study the presence of viral RNA was reported only in 23.8% of phlebovirus positive cases, probably due to the late collection of samples. In contrast, IgM and IgG for phleboviruses were detected by IIFT in 76.2% of the cases, in the presence of a negative molecular test. Interestingly, the outbreak reported in the Lombardy region in 2013 involved 14 patients out of 19 with no history of travel in endemic areas, suggesting the local acquisition of the infection and a spread of phlebovirus activity in new unexpected areas.

Of note, data obtained from entomological surveillance in the “Oltrepo’ Pavese area revealed the presence of different phleboviruses in collected sandflies [19]. It could be speculated that summer 2013 represented a favorable season for sandflies to provoke recrudenscence in those areas in which phleboviruses do not normally circulate. Furthermore, the northward spread of leishmaniosis in Italy [20] indirectly confirms the spread of both the phlebotomine vectors and phleboviruses in hilly and low mountain areas of several continental regions, including the Lombardy region [21]. Thus, these data show the circulation of phleboviruses in Northern Italy, beyond Central and Southern Italy. For these reasons, public health authorities should take into account the risk of outbreaks in Northern Italy, and implement appropriate preparedness plans, including vector control strategies.

A significant number of novel sandfly-borne phleboviruses has also been discovered as a result of the recent growth in interest in this group. The high number of different viruses in this group is a consequence of the high mutation rates, which result in the accumulation of point mutations, a situation favoring the development of new mutants [2]. Moreover, the reassortant capacity of these viruses contributes to this diversity, as highlighted in the case of Ponticelli I, II, and III viruses. Interestingly, no cross-protection was observed between different strains, thus exposing patients to multiple infections from phleboviruses [22]. The results obtained by neutralization assay in our positive cases suggest the circulation of different phleboviruses without cross-neutralization and, consequently, probable absence of cross-protection. The analysis of the Nt Abs results shows that only one of the three Ponticelli strains reacts with some patient’s sera, suggesting that the reassortment of the M segment in phleboviruses is extremely important in the serological evaluation. Therefore, in some of our cases, no Nt Abs were detected with the strains used in the neutralization assay, thus confirming this hypothesis.

One of the major limitations of the current study was the lack of a second serum sample as a follow-up for further serological tests for many of the analyzed patients. Therefore, we were not able to detect a seroconversion to confirm the diagnosis. Moreover, our patient population showed different levels of symptoms, ranging from mild fever to neurological involvement, probably because of circulation of different strains. However, due to the low rate of positive molecular results, we were not able to corroborate this hypothesis. The possibility that the pathogenic virus was not yet isolated, and our use of only an affine virus in our assay cannot be discarded as explanations because of the high phlebovirus diversity, also recorded in the study area, and the failure to isolate phleboviruses detected in sandflies. It is conceivable that during the period of our study, many cases of meningitis and encephalitis occurring during the warm months remained undiagnosed. To date, phlebovirus infections have been inadequately considered by physicians and are generally underestimated. However, given our results, Sandfly fever viruses should be routinely included in diagnostic panels during warm months, including in Northern Italy.

## Figures and Tables

**Table 1:** Pattern of molecular and serological results in 649 tested subjects.

	IgM+/IgG−	IgM+/IgG−/+ NT Abs−/+	IgM−/IgG− NT Abs−	Total Number of Patients
TOSV RT-PCR/ Panphlebo+	1	9	0	10
TOSV RT-PCR/ Panphlebo−	0	32	235	267
TOSV RT-PCR/ Panphlebo ND	0	0	372	372
Total	1	41	607	649

Abbreviations: TOSV: Toscana virus; RT-PCR: retro-trascriptase polymerase chain reaction; Panphlebo: panphlebovirus RT-PCR; NT Abs: neutralizing antibodies; ND: not done. “+”: positive; “−”: negative; “+/−”: positive or negative.

**Table 2:** Phlebovirus outbreak (summer 2013) in 19/51 patients in Lombardy region.

ID	Follow-Up	Age	Area	IFT Sandfly Fever Virus Mosaic 1 IgM		IFT Sandfly Fever Virus Mosaic IgG		NT Abs	RT-PCR		Travel in Endemic Area
				Serum	CSF	Serum	CSF		Serum	CSF	
1	A	52	Pavia	+	NA	−	NA	−	−	NA	Marche
5	A	38	Varese	+	NA	−	NA	−	−	NA	Greece
7	A	62	Pavia	+	NA	+	NA	−	−	NA	NO
8	A	59	Pavia	+	-	+	−	−	−	−	Calabria
10	A	66	Cremona	+	NA	+	NA	+	−	NA	NO
11	A	68	Cremona	+	NA	−	NA	−	−	NA	NO
13	A	89	Cremona	+	NA	−	NA	−	−	NA	NO
2	NA	85	Pavia	+	NA	−	NA	−	−	NA	NO
3	NA	65	Pavia	+	−	−	−	−	−	−	NO
4	NA	40	Brescia	+	−	−	−	−	−	−	Romania
6	NA	73	Pavia	+	−	+	−	−	−	−	Liguria
9	NA	98	Pavia	+	−	+	−	−	−	−	NO
12	NA	23	Cremona	+	−	+	−	−	−	−	NO
14	NA	89	Cremona	+	NA	+	NA	+	−	NA	NO
15	NA	85	Cremona	+	NA	+	NA	+	−	NA	NO
16	NA	37	Cremona	+	NA	+	NA	+	−	NA	NO
17	NA	48	Cremona	+	NA	−	NA	−	−	NA	NO
18	NA	35	Cremona	+	NA	+	−	+	−	−	NO
19	NA	66	Pavia	+	NA	+	NA	−	−	−	NO

Abbreviations: CSF: cerebrospinal fluid; Nt Abs: neutralizing antibodies; A: available; NA: not available; ND: not done; NO: no travel in endemic area; “+”: positive; “−”: negative.

**Table 3:** Neutralizing antibodies titer against four phlebovirus strains in 7/19 positive patients from the 2013 outbreak.

Patient ID	Serology Results				NT Abs Titer			
	Day of Sample Collection	Type of Phlebovirus Identified	IgM	IgG	181135/14 TOSV	181135/4 Ponticelli I	181135/8 Ponticelli II	220116/5 Ponticelli III
1	22 August 2013 6 September 2013	TOSV	P P	N P	<10 <10	<10 <10	<10 <10	<10 <10
5	13 September 2013 2 October 2013	SFCV	P P	N P	<10 20	<10 <10	<10 <10	<10 <10
7	21September 2013 2 October 2013 21 October 2013	SFNV	P P P	N P P	<10 <10 <10	<10 <10 <10	<10 10 40	<10 <10 <10
8	19 September 2013 8 October 2013	SFCV	P N	P P	10 40	<10 <10	<10 <10	<10 <10
10	24 September 2013 29 October 2013	SFSV	P P	P P	<10 <10	<10 <10	<10 20	<10 <10
11	24 September 2013 17 October 2013	SFCV	P P	N P	<10 <10	<10 <10	<10 20	<10 <10
13	15 October 2013 19 November 2013	TOSV	P P	N P	<10 640	<10 <10	<10 20	<10 <10

Abbreviations: TOSV: Toscana virus; P: positive; <10: negative NT Abs titer.

## Data Availability

The data presented in this study are available on request from the corresponding author.
